# Megestrol acetate *versus* metronomic cyclophosphamide in patients having exhausted all effective therapies under standard care

**DOI:** 10.1038/sj.bjc.6605623

**Published:** 2010-03-30

**Authors:** N Penel, S Clisant, E Dansin, C Desauw, M Dégardin, L Mortier, M Vanhuyse, F Bonodeau, C Fournier, J-L Cazin, A Adenis

**Affiliations:** 1 Centre Oscar Lambret, Lille, France; 2 Equipe d’Accueil 2694: Santé Publique, Epidémiologie et modélisation des maladies chroniques, Lille University, Lille, France; 3 Hôpital Saint-Vincent, Lille, France; 4 University Hospital, Lille, France

**Keywords:** palliative chemotherapy, megestrol acetate, metronomic cyclophosphamide, randomised phase II trial, sarcoma

## Abstract

**Background:**

To evaluate the antitumour activity and safety of metronomic cyclophosphamide *vs* megestrol acetate in progressive and advanced cancer patients having exhausted all effective therapies under standard care.

**Methods:**

Patients were randomly assigned to receive orally metronomic cyclophosphamide (50 mg b.i.d) or megestrol acetate (160 mg only daily) until intolerance or progression (RECIST 1.0). The primary efficacy end point was a 2-month progression-free rate (PFR_2m_). According to Optimal Simon's design and the following assumptions, namely, P0=5%, P1=20%, *α*=*β*=10%, the treatment is considered as effective if atleast 5 out of 44 patients achieved PFR_2m_.

**Results:**

Between September 2006 and January 2009, 88 patients were enrolled. Two patients experienced grade 3–4 toxicities in each arm (4%). One toxic death occurred in the megestrol acetate arm as a consequence of thrombosis. The metronomic cyclophosphamide arm reached the predefined level of efficacy with a PFR_2m_ rate of 9 out of 44 and a PFR_4m_ rate of 5 out of 44. The MA arm failed to achieve the level of efficacy with a PFR_2m_ of 4 out of 44 and a PFR_4m_ of 1 out of 44. The median overall survival was 195 and 144 days in the metronomic cyclophosphamide arm and megestrol acetate arm, respectively.

**Conclusion:**

Metronomic cyclophosphamide is well tolerated and provides stable disease in such vulnerable and poor-prognosis cancer patients. This regimen warrants further evaluations.

The care of cancer patients with a good performance status (PS) but having exhausted all available effective therapies under standard care represents a daily challenging situation for medical oncologists. Three possibilities may be discussed: exclusive palliative care, inclusion in phase I trials or treatment with a non-validated regimen, sometimes without a strong scientific basis. We know very well that a large proportion of such patients are not eligible for contemporary phase I trials because of an increasing number of eligibility criteria (Penel *et al*, 2008). Many patients with a good PS are reluctant to accept palliative care exclusively, and many medical oncologists are reluctant to propose exclusive palliative care in patients having a good PS. Nevertheless, the administration of an off-labeled chemotherapy regimen risks exposing patients to unnecessary toxicity without reasonable hope of clinical benefit, and unduly raises the cost of care. Considering these facts and the common nature of this situation, we explored different possibilities of treatment and retained metronomic cyclophosphamide and megestrol acetate as valuable options warranting further evaluation.

Metronomic chemotherapy refers to the frequent administration of chemotherapy, often daily, with no prolonged drug-free breaks, at doses significantly lower than the maximum tolerated dose (Kerbel and Kamen, 2004). One of the most frequently explored drugs for such uses is cyclophosphamide. In preclinical models, the metronomic administration of this bifunctional alkylating agent showed its ability to inhibit angiogenesis by inducing pericytes and endothelial cell dysfunction and apoptosis (Browder *et al*, 2000; Pietras and Hanahan, 2005; Yap *et al*, 2005). In retrospective series or clinical studies, metronomic cyclophosphamide alone or in combination provided some evidence of efficacy in several types of cancers, such hormone-refractory prostate cancer (Glode *et al*, 2003; Lord *et al*, 2007), heavily pretreated sarcoma (Casanova *et al*, 2004; De Pas *et al*, 2004; Stempak *et al*, 2006), ovarian cancer (Garcia *et al*, 2008) or breast cancer (Colleoni *et al*, 2006; Dellapasqua *et al*, 2008).

Megestrol acetate is currently used to improve appetite and increase weight in cancer-associated anorexia–cachexia syndrome (Mantovani *et al*, 1998; Desport *et al*, 2000; Berenstein and Ortiz, 2005; Yavuzsen *et al*, 2005). In 1993, the US Federal Drug Administration approved megestrol acetate for the treatment of anorexia, cachexia or unexplained weight loss in patients with AIDS. The mechanisms by which megestrol acetate increases appetite is largely unknown, but some data suggest an action on the pro-and anti-inflammatory interleukin network, especially a reduction in circulating tumour necrosis factor-*α* (Mantovani *et al*, 1998). A large meta-analysis had recently shown its ability to improve appetite and weight gain in cancer patients (Berenstein and Ortiz, 2005), and one randomised clinical trial showed its ability to improve the quality of life (Westman *et al*, 1999). Nevertheless, some studies had also pointed out the risk of phlebitis and pulmonary embolism related to MA (Rowland *et al*, 1996; Loprinzi *et al*, 1999). Furthermore, megestrol acetate had provided anecdotal objective responses for some hormone-independent solid tumours (Ravaud *et al*, 2008).

We carried out a multicentre randomised phase II trial to evaluate the safety and efficacy of metronomic cyclophosphamide *vs* megestrol acetate in cancer patients with a good PS and having exhausted all available effective treatments under standard care.

## Patients and methods

### Inclusion criteria

Patients were eligible if they had biopsy-proven cancer, were at least 18 years of age, had a good PS (⩽2), had exhausted all effective or validated therapies under standard care (chemotherapy, immunotherapy, molecular-targeted therapy and hormonal therapy), had progressive and measurable disease (according to Response Evaluation Criteria in Solid Tumour (RECIST 1.0) (Therasse *et al*, 2000)) before inclusion and were using effective contraception. They had to be able to swallow.

### Exclusion criteria

Patients excluded from the study were those who had had hypercalcaemia, breast cancer or low-grade stromal endometrial sarcoma, previous history of thrombosis or pulmonary embolism, dysphagia or malabsorption, neutrophil count <1500 mm^–3^, uncontrolled underlying comorbid disease, or any condition or underlying comorbid disease that may alter compliance. Pregnant or breastfeeding women were not eligible.

### Randomisation and site coordination

Patients were randomly (1 : 1) assigned to treatment with megestrol acetate or metronomic cyclophosphamide after registration through the Centre Oscar Lambret Clinical Research Unit. We used the method of random permuted block for randomisation.

### Treatment plan

Patients received both treatments until progression (RECIST 1.0) or intolerance, as complementary treatment of best supportive care. There was no planned dose modification. In the metronomic cyclophosphamide arm, as previously reported (Suvannasankha *et al*, 2007), treatment consisted of cyclophosphamide 50 mg b.i.d orally. In the acetate megestrol arm, the treatment consisted of acetate megetrol 320 mg once daily orally, as previously reported (Gebbia *et al*, 1996; De Conno *et al*, 1998; Westman *et al*, 1999; Yavuzsen *et al*, 2005).

### Study end points and data analysis

The primary efficacy end point was the progression-free rate (PFR) at 2 months. Secondary end points were PFRs at 4 and 6 months, toxicity according to the National Cancer Institute Common Toxicity Criteria (Version 3.0), overall survival and median time to progression, quality of life and rate of stable weight.

Consequently, during the study, patients underwent clinical and haematological evaluations at days 1, 15, 30 and 60, and every 2 months thereafter. Disease was assessed by comparing unidimensional tumour measurements (CT scan or MRI) on pre- and per-treatment imaging studies at 2, 4 and 6 months. We assessed response according to the RECIST 1.0. An independent third-party radiologist panel reviewed imaging studies to verify all imaging procedures carried out during the period of treatment with the trial drug, to ensure consistent unbiased application of RECIST 1.0. We defined ‘stable weight’ as weight loss <10% in comparison with baseline weight. Patients were surveyed at baseline, day 30 and day 60 using an auto-questionnaire (EORTC QLQC30 core questionnaire (Aaronson *et al*, 1993)). For all parameters, we carried out an intent-to-treat analysis.

### Statistical considerations

The number of patients was initially calculated using an ‘Optimal Simon's Design’ two-stage design (P0=5%, P1=20%, *α*=*β*=10%). Planned inclusion was 44 patients per arm. This design allowed the opening of the second stage if the PFR at 2 months was at least 1 out of 12. At the end of the second stage, if the PFR at 2 months was at least 5 out of 44, the treatment was defined as efficient.

Description of the populations used in the study was based on percentages and their 95% confidence intervals (95% CI) for categorical data, and median and extreme values for continuous data. The Kaplan–Meier method was used to calculate the median progression-free and overall survivals and their 95% CI.

The phase II randomised design did not allow a formal comparison between both arms.

### Ethical considerations

Study investigations were conducted after approval by the regional ethics committee (‘Comité de Protection des Patients Nord-Ouest III’, date of approval) and after declaration to the French Health Products Safety Agency (‘Agence Française de Sécurité Sanitaire et des Produits de Santé’, date of approval: June 2006). Informed consent was obtained from each patient. This study was registered in the European Clinical Trials Register (EudraCT No2006-003074-10, June 2006). The study was conducted in agreement with the Declaration of Helsinki and the International Conference on Harmonisation of Good Clinical Practise guidelines.

## Results

### Patient characteristics

Between September 2006 and January 2009, we enrolled 88 patients. Baseline characteristics were well balanced in both arms (Table 1). All patients experienced progressive disease before inclusion in the study. The median age was 66 years (CI: 57–71) in the megestrol acetate arm and 61 (CI: 50–72) in the metronomic cyclophosphamide arm. The median time between tumour diagnosis and inclusion was 27 months (CI: 18–36) in the megestrol acetate arm and 33 (CI: 24–42) in the metronomic cyclophosphamide arm.

### Toxicities

Table 2 describes all drug-related toxicities. Grade 3 and 4 toxicities were observed in the same range in both arms (4%, (CI: 1–6)). We observed a toxic death in the megestreol acetate arm: a case of Budd–Chiari syndrome (hepatic venous outflow obstruction) in a patient with massive liver metastasis from uveal melanoma. In the metronomic cyclophosphamide arm, we observed interstitial pneumonia in a patient with peritoneal metastasis from retroperitoneal liposarcoma, experiencing stable disease after 6 months of treatment. The most frequent side effects were oedema or hormonal and metabolic disorders in the megestrol acetate arm, and nausea, vomiting or anaemia in the metronomic cyclophosphamide arm. There was neither dose reduction nor transient treatment break in both arms. In the metronomic cyclophosphamide arm, one patient discontinued treatment for toxicity (pneumonitis at 6 months). In the megestrol acetate arm, two patients discontinued treatment for toxicity (phlebitis).

### Efficacy

**Figure 1:**
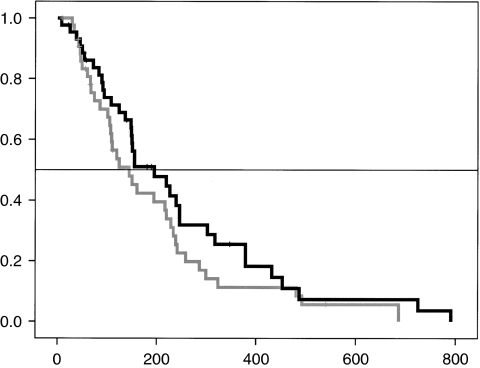
Overall survival (in days). Black line: metronomic cyclophosphamide, grey line: megestrol acetate.

At the end of the first stage, both treatments achieved statistical requirement, allowing the commencement of the second stage (at least one non-progression at 2 months among 12 patients). The megestrol acetate arm failed to reach the desired threshold of efficacy (Table 3). On the other hand, the metronomic cyclophosphamide arm successfully achieved the pre-defined level of efficacy with a PFR at 2 months of 9 out of 44 (20%, (8–32)). In total, six patients experienced stable disease at 4 months: one patient with metastatic limb liposarcoma treated with megestrol acetate, three patients with soft tissue sarcoma treated with metronomic cyclophosphamide, one patient with metastatic squamous cell skin cancer treated with metronomic cyclophosphamide and one patient with renal cell cancer treated with metronomic cyclophosphamide. The median duration of treatment was 57 days (52–61) with megestrol acetate and 58 (54–61) with metronomic cyclophosphamide. The median time to progression was 60 days (59–61) in both arms. Quality of life (Table 4) and weight stabilisation were similar in both arms. The median overall survival was 144 days (82–200) in the megestrol acetate arm and 195 (102–287) in the metronomic cyclophosphamide arm (Figure 1).

## Discussion

We evaluated the efficacy and toxicity of two treatments administered in cancer patients with a good PS and having exhausted all available therapies under standard care. This randomised clinical trial was ethical and conducted taking into account clinical equipoise. Both treatments (metronomic cyclophosphamide and megestrol acetate) gave comparable results in term of secondary end points. Nevertheless, acetate megestrol was responsible for the most severe side effects. Furthermore, metronomic cyclophosphamide was the sole treatment that could reach the predefined primary efficacy end point with a PFR at 2 months of 9 out of 44 and a PFR at 4 months of 4 out of 44.

Megestrol acetate did not reach the primary efficacy end point. Moreover, the rate of patients with stable weight was similar in both arms. Moreover, we had observed that acetate megestrol was the sole treatment responsible for oedemas that could overestimate the weight stabilisation. Acetate megestrol had been associated with compulsive eating and hypertriglyceridemia as a consequence of its orexigen effect. We had observed thrombotic events in two cases in this relatively small sample of patients. One of these thrombotic events led to death. Considering all these facts, we did not recommend administering megestrol acetate in such a population.

This study had some limitations. The choice of acetate megestrol as an internal comparator could be discussed, as endocrine tumours have been excluded (breast cancer, low-grade stromal endometrial sarcoma). Nevertheless, acetate megestrol is commonly administered in this patient population to maintain appetite. Moreover, rare responses have been reported with such hormonotherapy in patients with renal cancer and melanoma (Ravaud *et al*, 2008). There is no consensual dose for megestrol acetate (from 160 to 480 mg per day) or oral cyclophosphamide (more usually 50 mg per day) is this population. Nevertheless, Gebbia *et al* have shown that 480 mg of megestrol acetate was not superior to 320 mg (Gebbia *et al*, 1996). The dose of 50 mg b.i.d of cyclophosphamide has been administered in a previous phase II trial (Suvannasankha *et al*, 2007) in combination with thalidomide and prednisone without significant toxicity. The study population was a mix of patients with different tumours. The characteristics of the study population were similar to those of patients enrolled in phase I trials, with a 90-day mortality of approximately 20% (Italiano *et al*, 2008; Penel *et al*, 2008; Arkenau *et al*, 2009). In the current area of molecular-targeted therapies, one could argue the very low level of evidence to investigate both treatments in non-selected patients. Nevertheless, this study was pragmatic and addressed a very common, daily clinical issue. We believe that metronomic chemotherapy, especially metronomic cyclophosphamide, has been underevaluated. Many preclinical data and some clinical evidence suggest that this treatment inhibits angiogenesis (Bocci *et al*, 2003; Damber *et al*, 2005; Pietras and Hanahan, 2005; Yap *et al*, 2005), and some biological markers (circulating vascular endothelial growth factors, circulating endothelial cells, thrombospondin and so on) warrant further investigations as potential predictive factors (Bocci *et al*, 2003; Kieran *et al*, 2005; Munoz *et al*, 2005; Dellapasqua *et al*, 2008). Some could argue that this study did not formally establish the superiority of metronomic cyclophospahmide over acetate megestrol. Nevertheless, the purpose of randomised phase II trial is not to test such hypothesis but to show in parallel the results of both treatment arms (Cannistra, 2009). The ‘best’ arm is still chosen on the basis of the predetermined tumour size, as previously carried out in single-arm phase II trials. Randomisation minimises some pitfalls inherent to single-arm phase II trials, especially selection biases. Thus, the phase II randomised design leads to a double go–not go decision. The results of this study are sufficiently consistent to stop the evaluation of acetate megestrol in such a population and to favour new studies with metronomic cyclophosphamide in the same population. This study presents two strengths. All patients included experienced progressive disease before inclusion. Therefore, it is unlikely that the high rate of tumour stabilisation observed with metronomic cyclophosphamide was a spontaneous event. The second strength was the third-part CT-scan review that confirms stable diseases.

Eight heavily pretreated patients with sarcoma received metronomic cyclophosphamide in this study. We observed a 4-month non-progression rate of 3 out of 8. One patient experienced a stable disease for more than 30 months. The observed 4-month non-progression rate is in the range of results defining a second-line treatment as effective according to the EORTC recommendations (⩾40%) (Van Glabbeke *et al*, 2002). A previous rat model study had shown promising antitumour activity of metronomic cyclophosphamide administration in lymphoma and sarcoma (Rozados *et al*, 2004). De Pas *et al* had reported their single-centre experience of combined metronomic chemotherapy (cyclophosphamide plus methotrexate) in 17 heavily pretreated sarcoma patients. The median time of treatment was 3 months (range, 2–13), no grade 3–4 toxicity was noticed. Eight patients experienced stable disease (median time to progression 4 months, range 4–47+). Out of these eight patients, five experienced a progressing disease at the time of study entry. Our findings, together with these previous data, justify a further evaluation of metronomic cyclophosphamide in sarcoma patients.

On the contrary, no stable disease at 2 months was observed in patients with gastrointestinal cancers that represent approximately a third of enrolled patients.

Oral metronomic cyclophosphamide can be safely used on a metronomic basis in such a population. The efficacy, low toxicity, low cost (<£ 0.1 per day) and ease of administration of this treatment justify further studies in patients having exhausted all available therapies under standard care. We suggest that this treatment may be used as an internal comparator in further randomised phase II trials testing new options in some situations without established or shared consensual therapy.

## Figures and Tables

**Table 1:** Baseline characteristics

	**Megestrol acetate**	**Metronomic cyclophosphamide**
*n*	44	44
		
*Gender*		
Men	29 (66%, (52–80))	27 (61%, (47–75))
Women	15 (34%, (20–48))	17 (39%, (24–53))
		
*Tumour types*		
Colon and rectum cancers	11 (25%, (12–38))	11 (25%, (12–38))
Lung cancers	8 (18%, (6–29))	7 (16%, (5–26))
Soft tissue sarcoma	9 (20%, (8–32))	8 (18%, (6–29))
Melanomas	5 (11%, (2–20))	4 (9%, (0–17))
Bladder cancers	4 (9%, (0–17))	3 (6, (0–14))
Gastric cancers	1 (2%, (0–6))	5 (11%, (2–20))
Hepatocarcinoma	2 (4%, (0–10))	1 (2%, (0–6))
Unknown primaries	2 (4%, (0–10))	1 (2%, (0–6))
Other tumours	2 (4%, (0–10))	4 (9%, (0–17))
		
*Metastasis*		
Liver	17 (38%, (24–53))	16 (36%, (22–50))
Lung	17 (38%, (24–53))	19 (43%, (28–57))
Extra-abdominal lymph nodes	9 (20%, (8–32))	10 (22%, (10–35))
Abdominal lymph nodes	5 (11%, (2–20))	5 (11%, (2–20))
Pleura	4 (9%, (0–17))	1 (2%, (0–6))
Adrenal gland	3 (6%, (0–14))	6 (13%, (3–23))
Peritoneum	9 (20%, (8–32))	2 (4%, (0–10))
Bone	3 (6, (0–14))	3 (6, (0–14))
Brain	1 (2%, (0–6))	1 (2%, (0–6))
		
*Previously treated by*		
Surgery	31 (70%, (57–84))	31 (70%, (57–84))
Radiotherapy	23 (52%, (37–67))	22 (50%, (35–64))
		
*Performance status*		
0	22 (50% (35–65))	24 (54% (39–69))
1	17 (38% (18–45))	17 (38% (24–53))
2	5 (12% (1–20))	3 (3% (0–14))
		
*Number of previous systemic treatment lines*		
0	0	1 (2%, (0–6))
1	12 (27%, (14–40))	9 (20%, (8–32))
2	20 (45%, 30–60))	21 (47%, (36–66))
3	8 (18%, (6–29))	3 (6%, (0–14))
4	1 (2%, (0–6))	5 (11%, (2–20))
⩾5	3 (6%, (0–14))	5 (11%, (2–20))

**Table 2:** Treatment-related toxicities

	**Megestrol acetate**					**Metronomic cyclophosphamide**				
Grade	1	2	3	4	5	1	2	3	4	5
Fatigue	2	0	0	0	0	1	1	0	0	0
Hot flashes	1	0	0	0	0	0	0	0	0	0
Nausea	0	0	0	0	0	1	1	0	0	0
Vomiting	0	0	0	0	0	1	0	2	0	0
Anorexia	0	0	0	0	0	1	0	0	0	0
Compulsive eating	1	0	0	0	0	0	0	0	0	0
Hyper-triglyceridemia	0	0	1	0	0	0	0	0	0	0
Epigastralgia	0	0	0	0	0	1	0	0	0	0
Diarrhoea	0	1	0	0	0	0	0	0	0	0
Aphtosis	0	0	0	0	0	1	0	0	0	0
Anaemia	0	0	0	0	0	3	0	0	0	0
Neutropaenia	0	0	0	0	0	1	0	0	0	0
Phlebitis	0	1	0	0	1	0	0	0	0	0
Gynecomastia	1	0	0	0	0	0	0	0	0	0
Galactorrhoea	0	0	1	0	0	0	0	0	0	0
Libido alteration	0	1	0	0	0	0	0	0	0	0
Dysuria	0	0	0	0	0	0	1	0	0	0
Interstitial pneumonia	0	0	0	0	0	0	1	0	0	0
Oedema	1	2	0	0	0	0	0	0	0	0
Total	5	5	2	0	1	10	4	2	0	0

**Table 3:** Efficacy outcomes

	**Megestrol acetate (*n*=44)**	**Metronomic cyclophosphamide (*n*=44)**
Progression-free rate at 2 months	4 (9%, (0–17))	9 (20%, (8–32))
Progression-free rate at 4 months	1 (2%, (0–6))	5 (11%, (2–20))
Progression-free rate at 6 months	1 (2%, (0–6))	2 (4%, (0–10))
90-day mortality	12 (27%, (14–40))	9 (20%, (8–32))
Stable weight at day 30	11 (25%, (12–38))	6 (13%, (3–23))
Stable weight at day 60	6 (13%, (3–23))	4 (9%, (0–17))
Stable QOL at day 30	22 (50%, (35–64))	20 (45%, (30–60))
Stable QOL at day 60	10 (22%, (10–35))	18 (41%, (26–55))

**Table 4:** Quality of live assessments (median and extreme values)

	**MA-arm baseline (*n*=44)**	**MA-arm at 30 days (*n*=40)**	**MA-arm 60 days (*n*=28)**	**C-arm baseline (*n*=44)**	**C-arm 30 days (*n*=42)**	**C-arm 60 days (*n*=27)**
Physical functioning	5 (0–10)	6 (0–10)	8 (0–10)	4 (0–10)	4 (0–10)	7 (0–10)
Role functioning	2 (0–6)	3 (0–10)	5 (0–9)	3 (1–5)	4 (0–10)	5 (0–10)
Emotional functioning	5 (1–9)	6 (0–9)	6 (0–9)	6 (2–10)	6 (2–10)	6 (2–10)
Cognitive functioning	3 (0–5)	4 (0–7)	4 (1–8)	3 (1–6)	4 (1–8)	4 (1–9)
Social functioning	2 (0–5)	4 (0–8)	6 (2–8)	3 (0–7)	4 (0–10)	5 (0–10)
Quality of life	7 (4–7)	5 (2–7)	4 (0–7)	7 (7–5)	6 (1–7)	5 (0–7)
Fatigue	5 (0–9)	7 (0–10)	8 (2–10)	5 (0–10)	6 (0–10)	8 (0–10)
Nausea/vomiting	6 (0–10)	6 (2–10)	6 (0–10)	7 (0–10)	7 (0–10)	6 (0–10)
Pain	3 (0–5)	4 (0–10)	6 (3–10)	3 (1–6)	4 (2–9)	5 (0–9)
Dyspnoea	5 (1–8)	5 (0–9)	4 (2–9)	4 (0–8)	4 (2–10)	4 (2–9)
Sleep disturbance	8 (2–9)	7 (2–9)	8 (3–10)	7 (5–10)	8 (2–10)	7 (0–10)
Appetite loss	3 (0–6)	4 (0–10)	4 (1–9)	4 (1–6)	4 (0–8)	5 (0–10)
Constipation	2 (0–6)	3 (0–8)	4 (2–10)	3 (0–5)	4 (0–9)	4 (0–7)
Diarrhoea	1 (0–3)	1 (0–5)	1 (0–8)	2 (0–3)	2 (0–7)	2 (0–5)
Financial difficulty	4 (0–10)	6 (1–10)	6 (1–10)	5 (0–9)	5 (0–8)	6 (0–8)

Abbreviations: C=cyclophosphamide; MA=megestrol acetate.

There is no statistical significant difference at baseline, at 30 and 60 days in both arms.
